# Visual and rapid detection of *Acinetobacter baumannii* by a multiple cross displacement amplification combined with nanoparticles-based biosensor assay

**DOI:** 10.1186/s13568-019-0754-0

**Published:** 2019-02-26

**Authors:** Xueqin Cheng, Jing Yang, Meifang Wang, Peng Wu, Qiong Du, Jinjuan He, Yijun Tang

**Affiliations:** 1 Department of Respiratory and Critical Medical, Taihe Hospital, Hubei University of Medicine, Hubei, China; 2 Department of Clinical Laboratory, Taihe Hospital, Hubei University of Medicine, Hubei, China; 3 Department of Pharmacy, Wuhan General Hospital of the Chinese People’s Liberation Army, Hubei, China

**Keywords:** *Acinetobacter baumannii*, Multiple cross displacement amplification, Lateral flow biosensor, MCDA-LFB, Detection limit

## Abstract

The traditional microbiological methods used for detecting *Acinetobacter baumannii* were usually time-consuming and labor-intensive. Thus, we sought to establish a novel rapid detecting method for target pathogen. A set of multiple cross displacement amplification (MCDA) primers was designed to recognize 10 different regions of the *pgaD* gene, which was conservative and specific for the bacterium. In the MCDA system, amplification primers D1 and R1 were 5′-labeled with FITC (fluorescein) and biotin, respectively. Numerous FITC- and biotin-attached duplex amplicons were formed during the amplification stage, which were detected by nanoparticles-based lateral flow biosensors (LFB) through immunoreactions (FITC on the duplex and anti-FITC on the LFB test line) and biotin/streptavidin interaction (biotin on the duplex and streptavidin on the nanoparticles). The results showed that the optimized reaction condition of MCDA-LFB method was 62 °C within 25 min. There was no cross reaction with non-*A. baumannii* species and the non-*Acinetobacter* genera, and the detection limit for DNA samples was 100 fg/reaction. For 135 sputum samples, the detection results showed that the detection ability of MCDA-LFB assay was superior to the culture methods and conventional PCR. Therefore, MCDA-LFB assay could be a potential tool for the rapid detection of *A. baumannii* in clinical samples and low resource areas.

## Introduction

*Acinetobacter baumannii* (*A. baumannii*), a strict-aerobic, gram-negative, rod-shaped, opportunistic pathogen, extensively exists in natural and medical environments (Bergongne-Berezin and Towner 1996; Van Looveren and Goossens 2004). In addition to responsible for respiratory tract infections and bacteremia, the organism also can cause urinary tract infection, secondary meningitis, peritonitis, and other types of infections, especially in immune-compromised patients and those patients who undergo invasive procedures (Marioni et al. 2010; Antunes et al. 2014; Zhou et al. 2014). Due to the lack of specific clinical symptoms and rapid diagnostic techniques, antibiotics in the early stage of *A. baumannii* infection can only be used experientially, which maybe overused or misused. As a result, a number of multidrug-resistant (MDR) *A. baumannii* strains appeared, even the emergence of extensively-drug resistant (XDR) and pandrug-resistant (PDR) strains that posed a difficult problem to clinical treatment, particularly in critically ill patients (Durante-Mangoni and Zarrilli 2011). Therefore, it is of vital to establish a rapid and accurate diagnosis method for guiding the appropriate therapy of *A. baumannii* in the early infection stage.

The gold standard used for diagnosis of *A. baumannii* infection is traditional culture-biochemical methods, which usually takes 24–72 h of incubation to produce positive results, and thus is typically labor intensive and time consuming. Consequently, this technique could not satisfy early detection of causative pathogens, which is crucial for the proper use of antimicrobial agents. Recently, a number of nucleic acid test methods were developed to detect and identify *A. baumannii.* PCR based on 16S–23S ribosomal DNA intergenic spacer region and *recA* gene were carried out to detect *A*. *baumannii* (Chiang et al. 2011). Real-time PCR systems based on *ompA*, *bla*_*oxa51*_, *bfs* and *bap* gene have also been used for its specific identification (Nomanpour et al. 2011; Zhang et al. 2011; McConnell et al. 2012; De Gregorio et al. 2015). In addition, Linear-after-the-exponential PCR based on *gyrB* gene has also been developed for confirming its specificity (Rice et al. 2013). However, these molecular methods require specialized high-cost equipment, which are not readily available in low-resource settings and outbreak control. Moreover, most of these genes could not effectively distinguish *A. baumannii* from *Acinetobacter pittii* (*Acinetobacter* genospecies 3), *Acinetobacter nosocomialis (Acinetobacter* genospecies 13TU) and *Acinetobacter lwoffii*, which have been recognized as important nosocomial pathogens recently (Dijkshoorn et al. 2007; Higgins et al. 2007; Turton et al. 2010). Thus, another rapid, simple and specific assay detecting the other target gene is required to replace current PCR assays.

Multiple cross displacement amplification (MCDA) method, a new nucleic acid amplification technique, which eliminated a thermocycler or sophisticated training, could be conducted at a constant temperature (60–67 °C) within 40 min (Wang et al. 2015). There are ten specific primers to recognize distinct regions of the target sequence. After termination of amplification, the gold nanoparticle-based lateral flow biosensors (LFB), a very suitable diagnosis technique, has been designed and applied to MCDA-amplified products detection (Wang et al. 2017).

In this study, MCDA technique coupled with LFB was developed for rapid and sensitive detection of *A. baumanni* at *pgaD* gene. *pgaD* is a specific and conservative sequences of *A. baumannii*, and encodes proteins that synthesize cell-associated poly-b-(1-6)-*N*-acetylglucosamine (PNAG) (Choi et al. 2009; Wang et al. 2013). In the study, ten specific primers targeting different sites of *A. baumannii*-*pgaD* gene were designed, and the condition of these primers in MCDA reaction was optimized. The specificity and sensitivity of the MCDA-LFB method for the detection of *A. baumanni* were evaluated. Finally, clinical samples of *A. baumannii* were identified by MCDA-LFB method, which was compared with the culture method and also with the conventional PCR method.

## Materials and methods

### Bacterial strains and genomic DNA preparation

A total of 43 bacterial strains were used in this study (Table 1). Species identification were carried out initially by using Gram’s method, microscopy, oxidase experiment, and further confirmed by DL-96 systems using 96E ID Card according to the manufacturers instructions. The identification results are showed automatically by accompanying software (culture and biochemical methods). Genomic DNA was extracted from the respective bacteria using a DNA Mini Kit, and extracted DNA was quantified with ultraviolet spectrophotometer (Nano drop one, Thermo, Beijing, China) at A260/280. *A. baumannii* strain ATCC19606 DNA was used for confirmation MCDA products, optimal amplification temperature, optimal detection time and sensitivity analysis, while DNA from *Klebsiella pneumoniae* strain ATCC700603 and *Pseudomonas aeruginosa* strain THH-PA001 were used as negative controls.

**Table 1:** Strains used and the results of MCDA assays

Bacteria	Strain no./source	No. of strains	MCDA-LFB result^b^
*Acinetobacter baumannii*	ATCC19606^a^	1	+
	Isolated strains from Taihe Hospital	20	+
*Acinetobacter nosocomialis*	Isolated strain	1	–
*Acinetobacter pittii*	Isolated strain	1	–
*Acinetobacter lwoffii*	Isolated strain	1	–
*Pseudomonas aeruginosa*	THH-PA001^c^	1	–
*klebsiella pneumoniae*	ATCC700603	1	–
*Haemophilus influenza*	Isolated strain	1	–
*Haemophilus parahaemolyticus*	Isolated strain	1	–
*Mycobacterium tuberculosis*	ATCC25177	1	–
*Neisseria meningitides*	ATCC13090	1	–
*Streptococcus pneumoniae*	Isolated strain	2	–
*Staphylococcus aureus*	Isolated strain	1	–
*Staphylococcus epidemidis*	Isolated strain	1	–
*Staphylococcus saprophyticus*	Isolated strain	1	–
*Vibrio cholera*	ICDC	1	–
*Salmonella*	ICDC	1	–
*Listeria monocytogenes*	ATCC51779	1	–
*Enterohemorrhagic E. coli*	ICDC	1	–
*Enteropathogenic E. coli*	ICDC	1	–
*Enterotoxigenic E. coli*	ICDC	1	–
*Enteroaggregative E. coli*	ICDC	1	–
*Enteroinvasive E. coli*	ICDC	1	–

^a^*ATCC* American type culture collection, *ICDC* National Institute for Communicable Disease Control Disease Control and Prevention, Chinese Center for Disease Control and Prevention

^b^+ Positive; − negative

^c^*THH* Taihe Hospital, *PA Pseudomonas aeruginosa*

### Reagents

QIAamp DNA Mini Kits and QIAamp DNA Microbiome Kits were purchased from Qiagen Co., ltd. (Beijing, China). The isothermal amplification kits, Lateral flow biosensors (LFB) and Malachite Green (MG) was purchased from BeiJingHaiTai-ZhengYuan Technology Co., Ltd. (Beijing). PCR MasterMix reagents were purchased from Tiangen Biotech Co., Ltd. (Beijing).

### Primer design

**Fig. 1:**
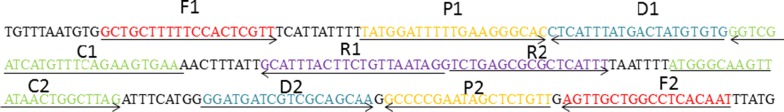
Schematic diagram of MCDA primer design for *pgaD* gene

A set of MCDA primers, including two displacement primers (F1 and F2), two cross primers (CP1 and CP2) and six amplification primers (C1, C2, D1, D2, R1 and R2), were designed based on the sequence of *pgaD* coding region by PRIMER PREMIER 5.0 and PrimerExplorer V4 (http://primerexplorer.jp/elamp4.0.0/index.html). All hybrids and hairpin structures of MCDA primers were analyzed by the Integrated DNA Technologies design tool (http://www.idtdna.com/site). Blast analysis was applied for confirming that all MCDA primers were specific for targeting *A. baumannii*. In order to detect MCDA products by LFB, the D1 and R1 primers were 5′-labeled with fluorescein (FITC) and biotin, respectively. The details of location, sequences and modifications of primers are listed in Fig. 1 and Table 2. All of the oligomers were synthesized and purified by TsingKe Biotech Co., Ltd. (Beijing, China) at HPLC purification grade.

**Table 2:** The primers used in this study

Primer	Sequence and modifications (5′–3′)	Length
F1	GCTGCTTTTTCCACTCGTT	19 nt
CP1	TCACTTCTGAAACATGATCGACCTATGGATTTTTGAAGGGCAC	43 mer
C1	TCACTTCTGAAACATGATCGACC	23 nt
D1	CACACATAGTCATAAATGAG	20 nt
D1*^a^	FITC-CACACATAGTCATAAATGAG	20 nt
R1	CCTATTAACAGAAGTAAATGC	21 nt
R1*^b^	Biotin-CCTATTAACAGAAGTAAATGC	21 nt
R2	TCTGAGCGCGCTCATTT	17 nt
D2	GATGATCGTCGCAGCAA	17 nt
C2	ATGGGCAAGTTATAACTGGCTTAG	24 nt
CP2	ATGGGCAAGTTATAACTGGCTTAGAACAGAGCTATTCGGGGC	42 mer
F2	ATTGTGAGGCCAGCAACT	18 nt

*nt* Nucleotide, *mer* monomeric

^a^D1*, 5ʹ-labeled with fluorescein (FITC) when used in the MCDA-LFB assay

^b^R1*, 5ʹ-labeled with biotin when used in the MCDA-LFB assay

### The MCDA assay

The MCDA reactions were carried out in a total of 25 μL reaction mixtures, and the amount of primer required for one reaction was 0.4 μmol L^−1^ displacement primers F1 and F2; 0.8 μmol L^−1^ amplification primers C1, C2, D1*, D2, R1*, and R2; 1.6 μmol L^−1^ cross primer CP1 and CP2; In addition, 1 μL *Bst* DNA polymerase (10 U), 12.5 μL 2XReaction Buffer, 1.5 μL Malachite Green and 1 μL of DNA template were also added to the amplification mixes. At the same time, 10 ng genome DNA of *klebsiella pneumoniae* ATCC700603 and *Pseudomonas aeruginosa* THH-PA001 were used as negative control, and 1 μL of double distilled water (DW) was used as blank control. Conventional PCR was performed as described previously (Wang et al. 2013).

Three monitoring methods, including colorimetric indicator (MG), gel electrophoresis and LFB, were used to analyze MCDA amplicons. When using MG, the amplified products cause a color change in the reaction solution from green to bright green, while the reaction solution of the negative controls and blank control without amplification turned colorless. In electrophoresis, a ladder-like band is observed for positive amplifications, but not in negative controls and blank control. By LFB, both the test line (TL) and the control line (CL) were observed for positive reactions, but only the CL was visualized in negative controls and blank control.

To validate the feasibility of MCDA primers, the reactions were initially incubated at 62 °C for 20 min and then inactived by heating at 85 °C for 2 min. To determine the optimal reaction temperature, eight distinct temperatures ranging from 60 to 67 °C at 1 °C interval were monitored in the Real-time Turbidimeter, and on the basis of the optimal reaction temperature, the MCDA mixture was incubated from 10 to 25 min with 5 min intervals to ensure the shortest time for LFB detection.

### Specificity and sensitivity of LFB assay for detecting *A. baumanni*-MCDA products

Genomic DNA of *A. baumanni* strain ATCC19606 diluted from 10 ng μL^−1^ to 1 fg μL^−1^ were prepared in DW to define the detection limit of MCDA-LFB assays. 1.0 μL DNA was used as template for per reaction, and each reaction was repeated at least twice. Detection of *A. baumanni*-MCDA products by LFB assay was compared to the methods of agarose gel electrophoresis and colorimetric indicator for three replicates.

Twenty-one *A. baumanni* strains and twenty-two non-*A. baumanni* strains were used to confirm the specificity of the MCDA-LFB assay. Each reaction was repeated three times.

### Application of MCDA-LFB assay in clinical sputum samples

To explore the clinical application value of the *A. baumanni*-MCDA-LFB assay, 135 clinical sputum samples were collected from Taihe (Shiyan, Hubei, China) hospitalized patients with cough or pneumonia and healthy people, which were first discriminated by the reference method, and then assessed by conventional PCR and MCDA-LFB established in this study. The reference method are the same as the above description of species identification. The PCR primer sequences used were: PgaD forward 5′-TGT TTA ATG TGG CTG CTT T-3′, and PgaD reverse 5′-CAG TCA TGG CTT AGT AAT AGT-3′. PCR reactions were conducted in a total of 20 μL volume containing 0.5 μM PgaD forward and PgaD reverse primer each, 10 μL 2XTaq PCR MasterMix II, 7 μL DW and 1 μL of DNA as the template. Reaction conditions were set at 93 °C for 3 min, followed by 35 cycles of 93 °C for 15 s, 53 °C for 15 s, 72 °C for 15 s, with a final extension at 72 °C for 5 min. The PCR products were then analyzed by 1.5% agarose gel electrophorsis.

## Results

### Determination of *A. baumanni*-MCDA products

**Fig. 2:**
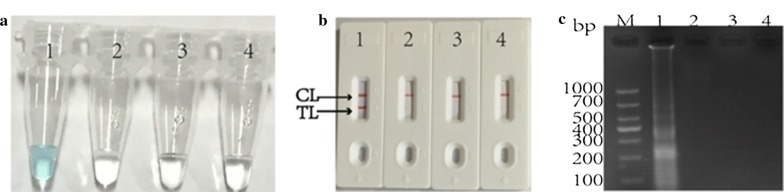
Determining of *A. baumanni*-MCDA products. **a** 1.5 μL of Malachite Green added to 25 μL of MCDA reaction mixture before the MCDA reaction was used to identify amplification products of the *A. baumanni*-MCDA assay; **b** Lateral flow biosensor (LFB) applied for visual detection of *A. baumanni*-MCDA products; **c** The *A. baumanni*-MCDA products were analyzed using agarose gel electrophoresis. Lane M, DNA maker DL1000. Tube/Biosensor/Lane: 1, positive amplification of *A. baumanni* strain ATCC19606; 2, negative control (*Klebsiella pneumoniae* strain ATCC700603); 3, negative control (THH-PA001); 4, blank control (DW). TL, test line. CL, control line

The effectiveness of the MCDA primers targeting *A. baumanni*-*pgaD* gene were evaluated by three groups at 62 °C for 20 min, including positive control (*A. baumanni*), negative control (*Klebsiella pneumoniae* and *Pseudomonas aeruginosa*) and blank control (DW). Three diagnosis methods were used to analyze MCDA products, such as Malachite Green, agarose gel electrophoresis and LFB. The reference strain ATCC19606 DNA produced positive results as shown in Fig. 2, the color of reaction mixture turned bright green at the end of amplification, two red bands were visible on the control line and the test line of a LFB, and a ladder-like band occurred on 2% gel electrophoresis. However, the DNA from *klebsiella pneumoniae* ATCC700603, *Pseudomonas aeruginosa* THH-PA001 or the blank control produced negative results as follows: (i) the reaction mixture without amplicons turned colorless, (ii) only a single red band appeared on the biosensor control line, (iii) no ladder-like bands arise. Therefore, these results indicated that the primer set designed in the current study was a good candidate for establishment of MCDA-LFB approach for *A. baumanni* detection.

### The optimal temperature assessed for MCDA assay

**Fig. 3:**
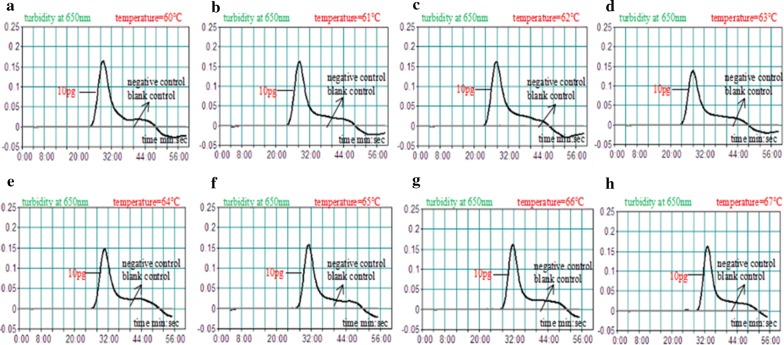
Optimization amplification temperature for *A. baumannii*-MCDA primers. MCDA reactions for detection of *A. baumannii* were monitored by real-time measurement of turbidity and the corresponding curves of DNA concentration were marked in the diagram. Turbidity of > 0.1 was considered positive. Kinetic curves (**a**–**h**) were generated at 60–67 °C (1 °C intervals) respectively with 1 pg DNA per reaction

In order to obtain the optimal temperature of the primers in the MCDA reaction, we adopted a real-time turbidity to monitor amplification state of the reference strain DNA at eight different temperatures (ranging from 60 °C to 67 °C at 1 °C interval) for 1 h. The result showed that the MCDA products at 62 °C displayed the highest level of amplified amount of *pgaD* gene with shortest time than that at other temperatures as observed in Fig. 3.

### The detection limit of *A. baumanni*-MCDA-LFB assays

**Fig. 4:**
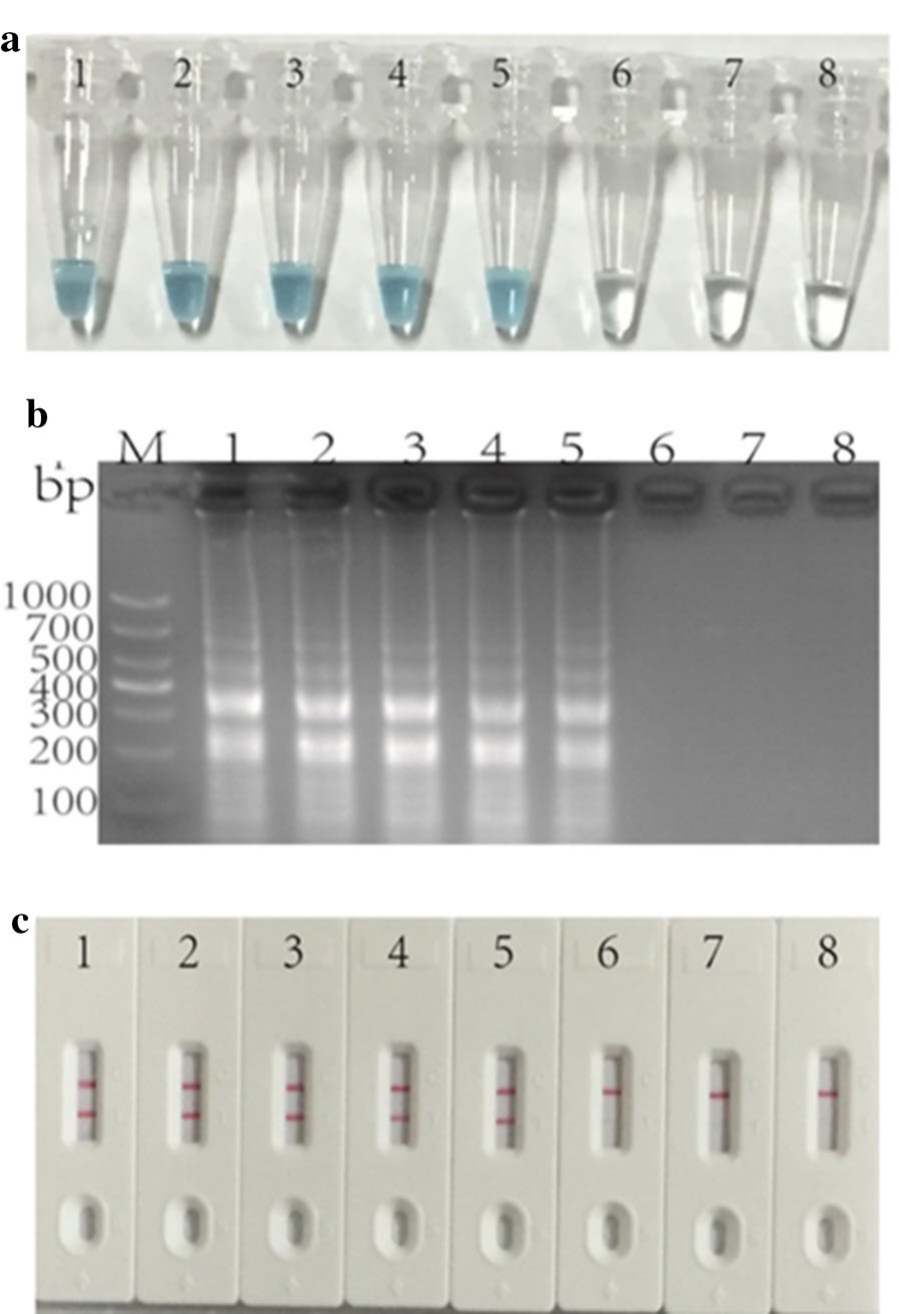
The detection limit of *A. baumanni*-MCDA-LFB assays. Three measurement techniques, including colorimetric indicator (**a**), gel electrophoresis (**b**) and lateral flow biosensor (**c**), were applied for analysis of MCDA amplicons. Serial dilutions of the template (10 ng, 100 pg, 10 pg, 1 pg, 100 fg, 10 fg and 1 fg) were subjected to standard MCDA reactions. Tubes (**a**), lanes (**b**), biosensors (**c**) 1–8 respectively represent *A. baumannii* strain ATCC19606 DNA levels of 10 ng, 100 pg, 10 pg, 1 pg, 100 fg, 10 fg and 1 fg per reaction, and a blank control (DW)

In order to know the detection limit of MCDA-LFB assay, eight different concentrations (ranging 10 ng μL^−1^ to 1 fg μL^−1^) of *A. baumanni* strain ATCC19606 DNA were respectively amplified at 62 °C for 20 min, and a red band appeared in the LFB test line with the detection limit being 100 fg μL^−1^ in 2 min. As observed in Fig. 4, the miniumu amount of Malachite Green assay or gel electrophoresis assay for *A. baumanni*-MCDA products was also 100 fg per reaction, which was in complete accordance with LFB detection.

### The optimal detection time of LFB assay for *A. baumanni*-MCDA products

**Fig. 5:**
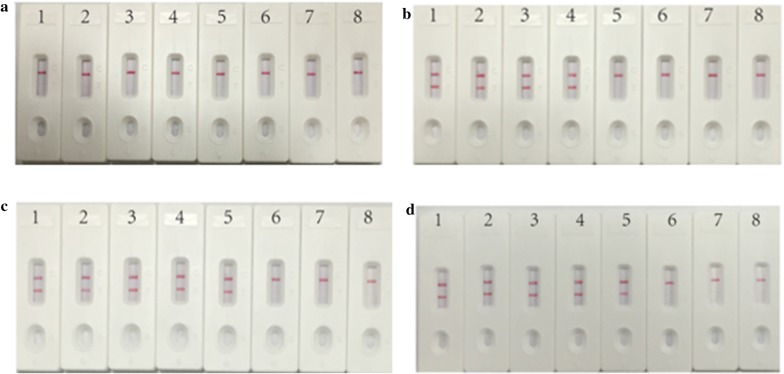
Optimal detection time required for *A. baumanni* MCDA-LFB assay. Four different reaction times (**a** 10 min; **b** 15 min; **c** 20 min; **d** 25 min) were evaluated at 62 °C. Biosensors 1–8 represent *A. baumanni* strain ATCC19606 DNA levels of 10 ng, 100 pg, 10 pg, 1 pg, 100 fg, 10 fg and 1 fg per reaction, and a blank control (DW), repectively. The best sensitivity was observed when MCDA lasted for 20 min (**c**)

As observed in Fig. 5, when MCDA lasted for 10 min, only the control line was observed in a LFB; When MCDA lasted for 15 min, only the amplified products of the initial DNA level of 1 pg above produced postive results; When MCDA amplified for 20 min and 25 min, the limit of detection were both 100 fg by LFB, so the optimal reaction time for LFB to detect the amplified products was 20 min.

### Specificity of LFB assays detecting *A. baumanni*-MCDA products

**Fig. 6:**
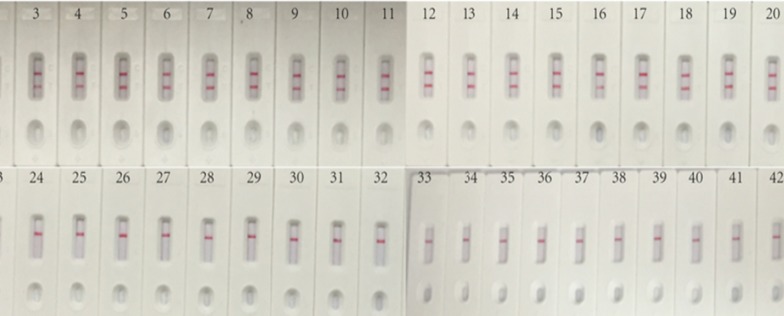
Specificity of LFB assays detecting *A. baumanni*-MCDA products. Different genomic DNA templates extracted from 43 strains were analyzed by MCDA-LFB assays. Two red lines were visuable in LFB for all *A. baumannii,* and only the control line was observed in non-*A. baumannii*. 1, Positive control (*A. baumannii* ATCC19606), 2–21, isolated *A. baumannii* strains. 22, *Acinetobacter nosocomialis*; 23, *Acinetobacter pittii*; 24, *Acinetobacter lwoffii*; 25, *Pseudomonas aeruginosa* THH-PA001; 26, *klebsiella pneumoniae* ATCC700603; 27, *Haemophilus influenza*; 28, *Haemophilus parahaemolyticus*; 29, *Mycobacterium tuberculosis* ATCC25177; 30, *Neisseria meningitides* ATCC13090; 31–32, *Streptococcus pneumoniae*; 33, *Staphylococcus aureus*; 34*, Staphylococcus epidemidis*; 35, *Staphylococcus saprophyticus*; 36*, Vibrio cholera;* 37, *Salmonella*; 38, *Listeria monocytogenes* ATCC51779; 39, *Enterohemorrhagic E. coli*; 40, *Enteropathogenic E. coli*; 41, *Enterotoxigenic E. coli*; 42, *Enteroaggregative E. coli*; 43, *Enteroinvasive E. coli*

To confirm the specificity of LFB assay for *A. baumanni*-MCDA products, DNA were respectively extracted from 21 *A. baumanni* strains and 22 non-*A. baumanni* strains, and then were amplified under the optimized conditions. As shown in Table 1 and Fig. 6, LFB assays for *A. baumanni*-MCDA products did not cause cross reaction with non-*A. baumannii* species and the non-*Acinetobacter* genera but *A. baumanni*, indicating that LFB assay was highly select and specific for *A. baumannii***-**MCDA products.

### Application of MCDA-LFB assay in clinical sputum samples

To further confirm whether MCDA-LFB assay could be applied in clinical samples, a total of 135 sputum samples, including 130 sputum samples collected from patients and 5 sputum samples collected from healthy controls, were detected. Of the 130 clinical sputum samples, MCDA-LFB assays detected 30 positive samples, which contain 26 sputum samples that have been confirmed positive by conventional PCR, and 22 confirmed by the culture assays. No positive results were determined from healthy controls (Negative control) by three detecting methods (Table 3). The MCDA-LFB assay showed 100% sensitivity, 92.9% specificity, 73.7% positive predictive value and 100% negative predictive value when compared to Culture-based assay. The MCDA-LFB assay showed 100% sensitivity, 96.3% specificity, 86.7% positive predictive value and 100% negative predictive value when compared to PCR assay.

**Table 3:** Comparison of methods for detection of *A. baumannii* in sputum samples

Detection methods	Sputum samples (n = 135)	
	Positive	Negative
Culture-based assay	22	113
PCR	26	109
MCDA-LFB assay	30	105

## Discussion

In the present study, a new amplification technique MCDA combined with the LFB assay was developed for rapid detection of *A. baumanni*. To date, the MCDA-LFB assay also had been successfully applied to the rapid detection of *Staphylococcus aureus, Klebsiella pneumoniae* and *Pseudomonas aeruginosa* (Wang et al. 2018a, b; Zhao et al. 2018). In addition to its high specificity, the MCDA-LFB assay could detect as low as 100 fg *A. baumannii* DNA, which was as sensitive as the *A. baumannii*–LAMP method, the limit detection of which was also 100 fg (Soo et al. 2013).

The detection methods used for analysis of MCDA products rely mainly on agarose gel electrophoresis, real-time turbidity, colorimetric indicator and LFB. The test of MCDA products by agarose gel electrophoresis needs an additional gel imaging system, which limits its application in low resource areas, and so does the expensive real-time turbidity. Another diagnosis method colorimetric indicator is somewhat subjective as the insufficient amplicons may lead to ambiguous judgment. Particularly, LFB avoids these disadvantages, easy to conduct, carry and the cost it takes only $US 2, eliminating the tedious gel electrophoresis, the use of hazardous EB and special devices, detecting the amplicons by observing whether the control line and the test line simultaneously appeared on the biosensor within 2 min. Hence, indicating MCDA result by LFB is objective, simple and rapid. In addition, electrophoresis, real-time turbidity and colorimetric indicator could not effectively differentiate specific amplicons from non-specific amplicons when MCDA generated a complex mixture of various amplicons, because a total of ten primers were employed for MCDA reaction system. In this report, we employed LFB for reporting the amplification results, which depends on anti-FITC on the LFB test line specially capturing FITC on the duplex. As a result, LFB analysis effectively overcomes this problem caused by non-specific DNA amplification, and further increases specificity on the basis of MCDA reaction. Therefore, LFB is more suitable for determining MCDA products.

The results of clinical specimens detection demonstrated that MCDA-LFB assays with the detection rate by 23.1% were more sensitive than conventional PCR of 20% and culture methods of 16.9%. The reason may be that culture assays could only be done in the situation of viable bacterial in the collected samples, so the death of strains would lead to false-negative results. However, DNA-based detection assays can avoid this problem, which is conducted in pathogen, alive or dead. Here, conventional PCR failed to amplify some samples that were confirmed positive by MCDA-LFB assays, may be due to lower amounts of bacterial templates. Moreover, the cost of each MCDA-LFB test is affordability with about $US 5.5, including MCDA per reaction with $US 3 and a LFB with $US 2. Besides, the only required instrument for the MCDA-LFB system is a constant temperature heater, such as a dry block heater with 1 kg, which is very portable, and in the whole operation process, no special training or a certified laboratory was required. Herein, the run cost declines.

In conclusion, we successfully developed a rapid, convenient, specific, sensitive, visual, free of special equipment and low cost MCDA-LFB assay for *A. baumannii* detection in clinical lower respiratory tract samples at 62 °C within 25 min getting rid of sample handling, which can be a valuable tool for surveillance and diagnosis of *A. baumannii* in the early infection stage, especially in routine detection, field use and resource constrained settings. Later, we will research on other clinical sample types using MCDA-LFB assay, including blood, urine, throat swabs and wounds, to further validate the practicability of the *A. baumannii* MCDA-LFB assay. To go a step further, we will study the rapid detection of drug resistance genes of *A. baumannii* on the basis of *A. baumannii* MCDA-LFB assay established in this study, to lay the foundation for developing a set of multi-channel rapid detection of multiresistant *A. baumannii*.
